# Supplementary material for: Animacy semantic network supports causal inferences about illness
Source: eLife. 2025 Nov 12;13:RP101944. doi: 10.7554/eLife.101944 (PMC12611283; doi:10.7554/eLife.101944)
Supplement: Supplementary file 1. [file elife-101944-supp1.docx]

Supplementary Table 1: Illness types present in the stimulus set.

| **Illness type** | **Number of trials** | |
| --- | --- | --- |
| acne | 2 |  |
| allergies | 1 |  |
| anemia | 1 |  |
| asthma | 1 |  |
| blood cancer | 1 |  |
| brain cancer | 1 |  |
| breast cancer | 1 |  |
| cancer (unspecified) | 1 |  |
| chickenpox | 1 |  |
| cold | 2 |  |
| COVID | 2 |  |
| epilepsy | 1 |  |
| flu | 3 |  |
| food poisoning | 1 |  |
| GI inflammation | 2 |  |
| GI virus | 1 |  |
| heart disease | 3 |  |
| high blood pressure | 1 |  |
| HIV-AIDS | 1 |  |
| liver disease | 2 |  |
| lung cancer | 1 |  |
| lung disease | 2 |  |
| malaria | 1 |  |
| pneumonia | 1 |  |
| skin cancer | 2 |  |
| throat cancer | 1 |  |
| Type 2 diabetes | 1 |  |
