# Supplementary material for: Animacy semantic network supports causal inferences about illness
Source: eLife. 2025 Nov 12;13:RP101944. doi: 10.7554/eLife.101944 (PMC12611283; doi:10.7554/eLife.101944)
Supplement: Supplementary file 2. — Each fROI was created by selecting the top 300 vertices for each contrast (see ‘Contrast’) in each search space. Accuracy refers to classifier performance against chance (50%) for Illness-Causal vs. Mechanical-Causal. Permuted and Bonferroni-corrected (across fROIs) p-values are reported. Ment_vs_phys: mentalizing stories>physical stories (mentalizing localizer). Caus_vs_rest: Illness-Causal+Illness-Mechanical>Rest. Logic_vs_lang: logic >language (language/logic localizer). Lang_vs_math: language>math (language/logic localizer). Visualizations of these results are displayed in Figure 1—figure supplement 11. [file elife-101944-supp2.docx]

Supplementary Table 2: Results of preregistered MVPA for *Illness-Causal* vs. *Mechanical-Causal* in individual-subject functional ROIs. Each fROI was created by selecting the top 300 vertices for each contrast (see ‘Contrast’) in each search space. Accuracy refers to classifier performance against chance (50%) for *Illness-Causal* vs. *Mechanical-Causal*. Permuted and Bonferroni-corrected (across fROIs) p-values are reported. Ment_vs_phys: *mentalizing stories* > *physical stories* (mentalizing localizer). Caus_vs_rest: *Illness-Causal + Illness-Mechanical* > *Rest.* Logic_vs_lang: *logic > language* (language/logic localizer). Lang_vs_math: *language > math* (language/logic localizer). Visualizations of these results are displayed in Supplementary Table 2 – Figure supplement 1.

| **Search space** | **Contrast** | **Accuracy** | **t** | **Permuted p** | **Bonferroni adj. p** |
| --- | --- | --- | --- | --- | --- |

| LTPJ | ment_vs_phys | 61.70% | 2.96 | 0.0053 | 0.032 |
| --- | --- | --- | --- | --- | --- |
| RTPJ | ment_vs_phys | 52.50% | 0.71 | 0.2573 | 1 |
| LPC | ment_vs_phys | 61.30% | 3.44 | 0.003 | 0.0112 |
| RPC | ment_vs_phys | 60% | 2.4 | 0.0176 | 0.108 |
| LTPJ | caus_vs_rest | 58.30% | 4.16 | 0.0004 | 0.0024 |
| RTPJ | caus_vs_rest | 51.30% | 0.39 | 0.3305 | 1 |
| LPC | caus_vs_rest | 60.80% | 2.94 | 0.0055 | 0.0336 |
| RPC | caus_vs_rest | 53.70% | 1.1 | 0.1516 | 1 |
| Logic | logic_vs_lang | 60.40% | 3.46 | 0.0017 | 0.0026 |
| Language | lang_vs_math | 58.80% | 2.76 | 0.0069 | 0.0124 |
