# Supplementary material for: Animacy semantic network supports causal inferences about illness
Source: eLife. 2025 Nov 12;13:RP101944. doi: 10.7554/eLife.101944 (PMC12611283; doi:10.7554/eLife.101944)
Supplement: Supplementary file 3. — Each fROI was created by selecting the top 300 vertices for each contrast in each search space: left PC (LPC)=top main experimental conditions>rest, language = top language>math (language/logic localizer), logic = top logic >language (language/logic localizer). Accuracy refers to classifier performance against chance (50%) for each test. Permuted and Bonferroni-corrected (across fROIs) p-values are reported. Visualizations of these results are displayed in Figure 1—figure supplement 12. [file elife-101944-supp3.docx]

Supplementary Table 3: MVPA results for all tests in select individual-subject functional ROIs. Each fROI was created by selecting the top 300 vertices for each contrast in each search space: left PC (LPC) = top *main experimental conditions* > *rest,* language = top *language > math* (language/logic localizer), logic = top *logic > language* (language/logic localizer). Accuracy refers to classifier performance against chance (50%) for each test. Permuted and Bonferroni-corrected (across fROIs) p-values are reported. Visualizations of these results are displayed in Supplementary Table 3 – Figure supplement 1.

| **Search space** | **Test** | **Accuracy** | **t** | **Permuted p** | **Bonferroni adj. p** |
| --- | --- | --- | --- | --- | --- |

| LPC | ill-causal vs. mech-causal | 61.30% | 3.44 | 0.0018 | 0.007 |
| --- | --- | --- | --- | --- | --- |
| LPC | ill-causal vs. noncausal1 | 51.70% | 0.43 | 0.3345 | 1 |
| LPC | ill-causal vs. noncausal2 | 58.30% | 3.45 | 0.0018 | 0.007 |
| LPC | mech-causal vs. noncausal1 | 56.20% | 1.66 | 0.0542 | 0.284 |
| LPC | mech-causal vs. noncausal2 | 62.50% | 3.81 | 0.0005 | 0.003 |
| Language | ill-causal vs. mech-causal | 58.80% | 2.76 | 0.0097 | 0.093 |
| Language | ill-causal vs. noncausal1 | 54.60% | 1.5 | 0.083 | 1 |
| Language | ill-causal vs. noncausal2 | 55% | 1.61 | 0.0675 | 0.93 |
| Language | mech-causal vs. noncausal1 | 52.90% | 0.85 | 0.1957 | 1 |
| Language | mech-causal vs. noncausal2 | 53.30% | 1.09 | 0.1609 | 1 |
| Logic | ill-causal vs. mech-causal | 60.40% | 3.46 | 0.0029 | 0.0195 |
| Logic | ill-causal vs. noncausal1 | 60.40% | 3.27 | 0.0029 | 0.03 |
| Logic | ill-causal vs. noncausal2 | 52.90% | 0.88 | 0.1928 | 1 |
| Logic | mech-causal vs. noncausal1 | 53.80% | 1.23 | 0.1121 | 1 |
| Logic | mech-causal vs. noncausal2 | 55.80% | 1.97 | 0.0425 | 0.4785 |
